# A hybrid and cost-efficient barcoding strategy for full-length 16S rRNA gene nanopore sequencing of environmental samples

**DOI:** 10.1186/s12864-026-13239-z

**Published:** 2026-08-10

**Authors:** Ida Romano, Edoardo Pasolli, Jean-Claude Walser, Valeria Ventorino, Sonja Reinhard, Giuseppina Magaraci, Olimpia Pepe, Natacha Bodenhausen

**Affiliations:** 1 Department of Agricultural Sciences, Division of Microbiology, University of Naples Federico II, Piazza Carlo di Borbone 1, Naples, Portici, NA Italy; 2 Task Force on Microbiome Studies, University of Naples Federico II, Naples, Italy; 3 Genetic Diversity Centre (GDC), ETH Zurich, Universitätstrasse 16, Zurich, Switzerland; 4 Department of Soil Sciences, Research Institute of Organic Agriculture FiBL, Ackerstrasse 113, Frick, Switzerland

**Keywords:** Oxford Nanopore Technologies, Full-length 16S rRNA sequencing, Hybrid barcoding, Environmental microbiome, Microbial community profiling, MinION, Cost-efficient barcoding, Bioinformatic workflow

## Abstract

**Background:**

Accurate species-level identification of bacteria in complex environmental samples is essential for applications in biotechnology, ecological monitoring, and clinical diagnostics. Short-read platforms such as Illumina frequently truncate the 16S rRNA gene, limiting taxonomic resolution. In this work, we applied Oxford Nanopore Technology (ONT) long-read sequencing to full-length 16S rRNA amplicon in samples from natural soil amended with lignocellulosic biomass and a simplified microbial community derived from cultures grown on selective and differential carboxymethyl cellulose (CMC)-based substrates, with the aim to evaluate the difference in performance between a real, complex community and a less complex system.

To reduce consumable costs, we substituted the standard ONT Barcoding kits with an in-house hybrid barcoding workflow. Specifically, PacBio PCR-based barcoding protocol was used for sample indexing, followed by library preparation using the ONT Ligation Sequencing Kit. This simplified approach retained compatibility with MinION and Flongle flow cells and supported accurate downstream demultiplexing while lowering barcode costs substantially. Additionally, a new bioinformatic workflow tailored to ONT data was implemented.

**Results:**

Overall, the hybrid protocol significantly reduced per-sample barcoding costs while preserving high sequencing quality and throughput. The sequencing run yielded over 5 Gb of quality-filtered data (Q-score ≥ 10). Furthermore, the new bioinformatic workflow allowed taxonomic assignment at the species level for 49.38% of annotated taxa, compared to just 4.59% using Illumina NovaSeq sequencing of the V3-V4 region. ONT also recovered 2.3 times more genera and 1.3 times more families. Although 16S rRNA gene sequencing often cannot distinguish between closely related species, particularly within taxonomically complex groups, in this work, full-length reads substantially improved both taxonomic resolution and database matching.

**Conclusions:**

These results show that full-length 16S rRNA sequencing with ONT, paired with a low-cost barcoding strategy, enhanced taxonomic resolution compared to short-read workflows. This approach also offers a scalable and cost-effective option for high-resolution microbiome profiling in research and applied settings.

**Supplementary Information:**

The online version contains supplementary material available at https://doi.org/10.1186/s12864-026-13239-z.

## Background

The advent of next-generation sequencing (NGS) technologies between 2004 and 2006 revolutionized genomics and microbial ecology by increasing sequencing throughput while reducing per-base costs [1]. First applied in metabarcoding studies in 2006 [2], these high-throughput sequencing technologies marked a turning point in the field of microbial ecology, becoming over time more cost-effective and widely used [3]. The “short-read” sequencing technologies, like Illumina, are extensively used to describe microbial communities in different environments. These technologies entail the massively parallel sequencing of short reads, in which millions of discrete sequencing operations take place simultaneously [4]. By definition, short-read technologies generate short fragments, so reconstructing long DNA regions requires computational assembly, which can be challenging in repetitive or low-complexity regions [4]. When used to describe microbial communities, they frequently fall short when it comes to species-level identification [3].

The inability to resolve species in complex matrices is a significant drawback, particularly in environments with diverse microbial populations [3]. To address these limitations, long-read sequencing technologies emerged in the early 2010s, enabling the direct sequencing of extended DNA fragments, including full-length marker genes (species/strain level) [5]. Compared to short read sequencing, long-read sequencing often requires fewer library preparation steps than short-read approaches and allows real-time sequencing of intact DNA molecules [4]. Nevertheless, long reads have disadvantages, such as increased costs and high DNA amount requirements [6]. Another early limitation of these technologies was the low accuracy of the reads. However, accuracy has greatly improved over time, mainly due to advances in sequencing chemistry and the introduction of R10.4 flow cells which improves read accuracy, as well as improved basecalling models [4, 7].

Pacific Biosciences (PacBio) and Oxford Nanopore Technologies (ONT) dominate the long-read market. PacBio’s latest platform, Revio, now generates Q40 HiFi reads with accuracies of up to 99.99%, offering industry-leading performance for applications requiring high-precision long reads, whereas ONT historically produced less accurate reads, although accuracy has substantially improved over time with advances in sequencing chemistry and basecalling, now reaching > 97–99% under optimal conditions, but with multiple devices operating at different scales, ranging from handheld sequencing devices (MinION) to high-throughput sequencers (PromethION) [3].

ONT enables real-time, long-reads sequencing, allowing for in-depth taxonomic classification at the species and even strain levels. Compared to Illumina, ONT not only offers longer read lengths, which are particularly useful for characterizing microbial diversity and identifying functional genes in complex samples, but also offers the biggest advantages of portability due to the small size and off-line functionality of the machines, allowing them to be used to perform DNA sequencing in situ, for example in isolated regions [8]. Previous studies demonstrated that ONT can significantly improve the resolution of microbial community profiling in complex matrices and environmental samples such as mock communities, building dust, and soils [9–11]. Furthermore, research comparing long-read and short-read for 16S/18S/ITS sequencing demonstrated that long reads perform better at the species and genus levels [12]. The increased resolution provided by long-reads improves 16S rRNA classification and reduces the amount of reads that cannot be classified or assigned to lower-level taxa [13]. Johnson et al. (2019) [14] demonstrated that full-length approaches are sufficiently accurate to resolve subtle nucleotide differences among intragenomic 16 S rRNA gene copies, highlighting that this variability should be explicitly accounted for in downstream analyses. Short-read sequencing is generally limited to genus-level resolution and may underestimate microbial diversity, whereas full-length sequencing captures a greater phylogenetic signal and improves taxonomic assignment [14, 15]. Indeed, whole 16S rRNA sequences from microbial “dark matter” may be identified through long reads with a greater classification reliability and resolution [13].

Sequencing platform choice depends not only on analytical performance but also on cost, throughput, and laboratory infrastructure. Using custom, in-house barcoding strategies could be a key step towards reducing ONT sequencing costs and enhancing its applicability to analysing microbial communities in complex environmental settings. An additional consideration is the extensive heterogeneity of available protocols and kits, which may lead to inconsistencies and complications for end users. This underscores the necessity for a streamlined and standardized protocol.

This research focuses on the application of ONT sequencing for species-level identification of bacteria in complex environmental samples, specifically lignocellulosic biomass residues and soil. Their complexity makes them suitable benchmarks for assessing the performance of ONT sequencing relative to traditional Illumina methods. Additionally, this study explores potentials for cost-saving by replacing ONT’s standard Rapid Barcoding Kit with an in-house barcoding protocol that enables up to 384 barcodes combinations - notably higher than 24 or 96 barcodes offered in ONT’s commercial kits. Specifically, we have implemented a hybrid approach that uses PacBio-compatible, PCR-based barcodes for sample indexing, followed by ONT library preparation using the Ligation Sequencing Kit. This approach aims to reduce per-sample consumable costs while maintaining compatibility with ONTs MinION and Flongle platforms.

## Materials and methods

### Sample collection and DNA extraction

DNA templates for sequencing were extracted from a complex matrix composed of soil mixed with lignocellulosic biomass, collected from the forest undergrowth, which included also decomposing leaf litter and woody debris, collected from botanical garden of Department of Agricultural Sciences (40°49′11″N 14°20′28″E, Portici, NA).

In addition, bacterial bulk samples were obtained by first performing serial dilutions of the biomass, followed by plating on selective and differential media containing carboxymethylcellulose (CMC) as sole carbon source to isolate cellulolytic bacteria [16]. Microbial cells (bulk plate) were then collected from the less diluted plates, where there was sufficient colony growth and it was not possible to isolate new bacterial strains, using a suitable volume of quarter strength Ringer’s solution (Oxoid, Milan, Italy).

For molecular analysis, total genomic DNA was directly extracted from lignocellulosic biomass mixed with soil samples as well as from the bulk of cells (bulk plate), obtained by suspending all the colonies developed on the CMC agar plate surfaces, using FastDNA SPIN Kit for Soil (MP Biomedicals, Illkirch Cedex, France) according to the manufacturer’s instructions.

For additional research purposes, less diluted CMC plates were used for the isolation of cellulolytic bacteria. From the isolation procedure, a total of 11 cellulolytic bacterial strains obtained and identified by 16S rRNA gene sequencing. Genomic DNA was extracted by boiling for 10 min and used as template for PCR amplification of the 16S rRNA gene region amplified using universal primers 27 F (5′-AGAGTTTGATCCTGGCTCAG-3′) and 1492R (5′-TACGGYTACCTTGTTACGACTT-3′) [17]. PCR products were purified and sequenced as reported by Ventorino et al., 2016 [18], and sequences were taxonomically assigned using BLAST (GenBank database). The assigned sequences were deposited in the GenBank database under the following accession numbers: PX777770, PX776785, PX777775, PX777776, FBB7AA, PX777787, PX777875, PX776699, and PX776818. These previously characterized isolates (not re-isolated in the present work) were used as a reference dataset to evaluate the taxonomic resolution and accuracy of ONT MinION-based 16S rRNA gene sequencing applied to bulk plate samples obtained from less diluted plates. Specifically, species detected in the bulk samples by MinION sequencing were compared with the known species composition derived from the isolated strains.

### Sequencing workflow

#### Sample preparation for long reads 16s rRNA region with ONT

**Fig. 1:**
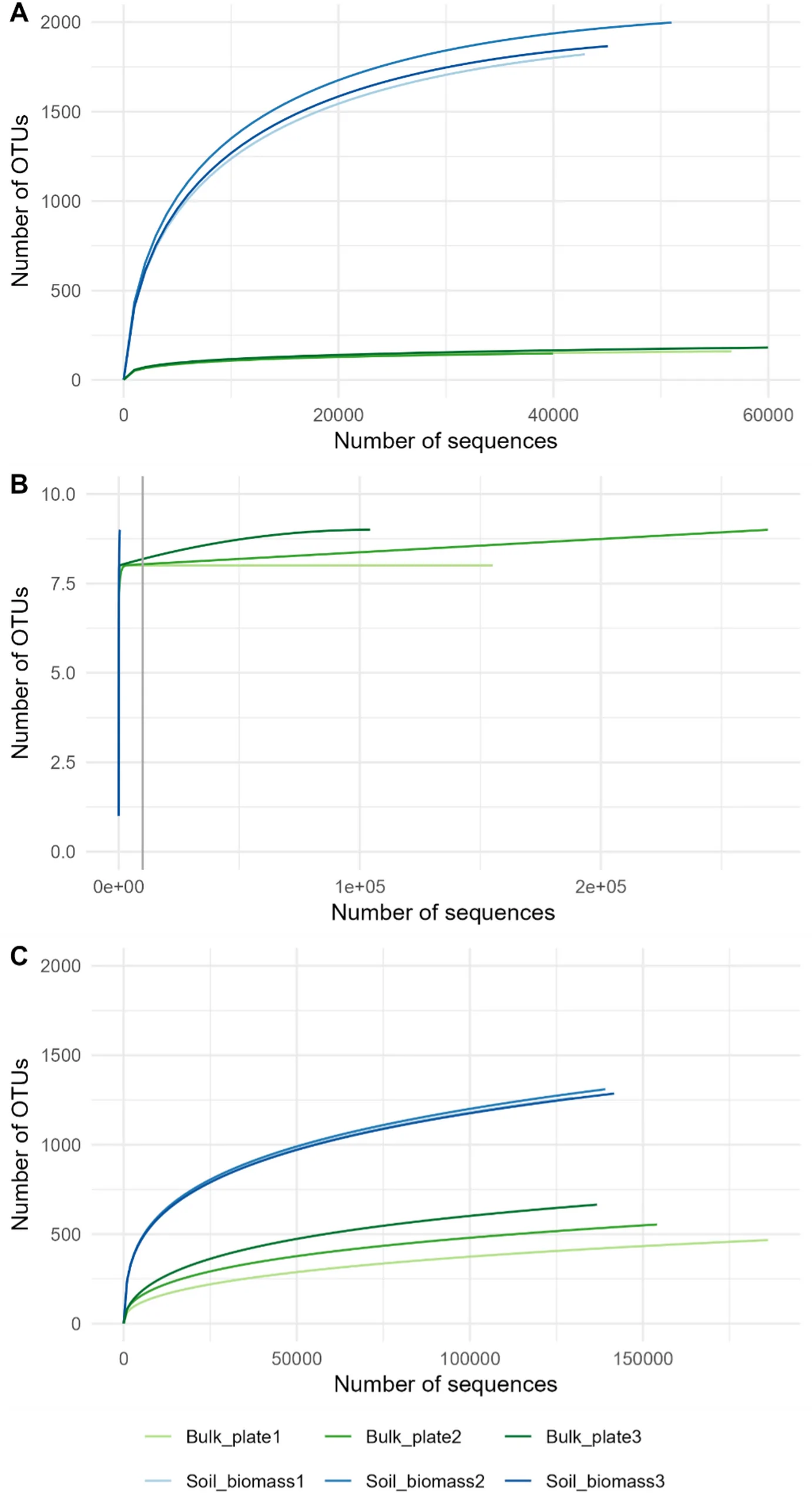
Rarefaction curves showing the number of observed OTUs as a function of sequencing depth for bulk plate (green) and soil-lignocellulosic biomass (blue) samples of (**A**) data obtained with Illumina sequencing, (**B**) data obtained from MinION sequencing and analysed with standard bioinformatic workflow (i), and (**C**) data obtained from MinION sequencing and analysed with tailored bioinformatic workflow (ii)

Indexed PCR products were generated using the PacBio Barcoded Universal Primers protocol using Barcoded M13 Primer Plate (https://www.pacb.com/wp-content/uploads/Procedure-checklist-Preparing-SMRTbell-libraries-using-PacBio-barcoded-M13-primers-for-multiplex-SMRT-sequencing.pdf). With a first PCR, the 16S region was amplified using universal primers 27F (5′-AGAGTTTGATCCTGGCTCAG-3′) and 1492R (5′-TACGGYTACCTTGTTACGACTT-3′) [17], both modified by adding a 5’ block and a M13 tail. In a 25 µl reaction, 12.5 µl of KAPA HiFi HotStart ReadyMix (2X) (Roche BIOSYSTEMS, R&D Cape Town, South Africa), 3 µl of 10 µM of each primer, 5.5 µl of water and 1 ul of DNA template (1 ng/ul ) were added. The PCR reactions were performed in strip tubes with individual caps to avoid cross-contaminations. The PCR program was 3 min at 95 °C, then 4 cycles of 20 s at 98 °C, 30 s at 57 °C and 1:30 min at 72 °C, followed by 35 cycles of 20 s at 98 °C, 30 s at 65 °C and 1:30 min at 72 °C, a final elongation step for 5 min at 72 °C on a CFX96 Touch Real-Time PCR Detection System (Bio-Rad Laboratories, Hercules, CA, USA). PCR products run on an agarose gel (1%) using the nucleic acid stain GelRed (Biotium, Fremont, CA, USA). Prior barcoding PCR, the PCR products were cleaned using Agencourt AMPure XP beads (Beckman Coulter™, Jersey City, NJ) and a 96 well Alpaqua^®^ magnetic plate. The cleaning was done by adding 0.8× volume of beads to each PCR product, followed by two washes with 200 µl of 70% ethanol. Dry beads were then resuspended in 25 µl of nuclease-free water. Negative PCR controls, consisting of all reaction components except template DNA, were included in each amplification run to assess potential contamination through gel electrophoresis (Supplementary Material 1 Fig. 1).

A second amplification was then performed to barcode the samples. In a 25 µl reaction, 12.5 µl of KAPA SYBR FAST Master Mix (2X) (Kapa Biosystems, Inc., USA), 3 µl of a unique combination of one forward and one reverse barcoded universal primer (each at 2.5 µM), and 1 µl of the product from the first-round PCR, which included a negative control. The PCR program was 3 min at 95 °C, then 2 cycles of 20 s at 98 °C, 15 s at 60 °C and 1:30 min at 72 °C, followed by 20 cycles of 20 s at 98 °C, 15 s at 65 °C and 1:30 min at 72 °C, a final elongation step for 5 min at 72 °C. All PCR products were checked on a gel as previously described and cleaned using AMPure XP beads (Life science).

The Ligation Sequencing Kit V14 (SQK-LSK114) from Oxford Nanopore Technologies was used to enable high quality DNA sequencing using the MinION device. Briefly, the protocol involves several steps including DNA preparation using 200 fmol of amplicon DNA, end-repair and dA-tailing, adapter ligation and purification prior to loading 20 fmol of the final prepared library onto the R10.4.1 flow cell. (FLO-MIN114).

The sequencing process using the MinION platform was conducted for a duration of 24 h. Basecalling was performed using Dorado v0.5.2 within MinKNOW v23.07.12, applying the basecalling model (SUP) provided by Oxford Nanopore Technologies. The quality of raw sequencing reads was evaluated using FastQC (version 0.12.0) to confirm the overall integrity of the sequencing data before proceeding with further downstream analyses.

#### Sample preparation and sequencing workflow for short-reads V3-V4 region

The same DNA underwent Illumina sequencing for comparison purposes with primers S-D-Bact-0341 F (5′-CCTAYGGGRBGCASCAG-3′) and S-D-Bact-0785R (5′-GGACTACNNGGGTATCTAAT-3′) [19] which amplify the V3-V4 region. Novogene prepared the library and performed the sequencing on their own machine. PCR conditions consisted of 25 cycles (95 °C for 30 s, 55 °C for 30 s, and 72 °C for 30 s) plus one additional cycle at 72 °C for 10 min as a final chain elongation. The PCR products of the correct size were evaluated using agarose (1.5%) gel electrophoresis. An equal amount of PCR products from each sample was then pooled, end-repaired and A-tailed before being ligated with Illumina adapters. The libraries were sequenced on an Illumina paired-end platform. The library was checked with AF2200 Plate Reader (Eppendorf, Milan, IT). Equimolar pools were obtained, and sequencing was carried out on Illumina NovaSeq 6000 platform at Novogene Bioinformatics Technology Co.

### Bioinformatics and sequence processing

#### Bioinformatics workflows for ONT sequences

Two distinct bioinformatics workflows were applied to analyse 16S rRNA sequencing data generated with MinION technology: (i) a standard workflow commonly used for paired-end Illumina short-read data, (ii) a workflow specifically tailored for processing ONT long-read sequences.

For workflow i), bioinformatics analysis was performed on the Scientific Computer Cluster Euler at ETH Zurich using USEARCH v11.0.667 following the protocol described by Romano et al. [20] Briefly, bioinformatic analysis was performed on the Euler cluster (ETH Zurich). Reads were processed using USEARCH v11, including phiX removal, primer trimming, quality filtering, and denoising. Chimeras were removed and sequences were clustered into ZOTUs using UNOISE, as well as into OTUs at 97% identity using UPARSE. Additionally, clustering at 97% sequence identity was done by UPARSE [21]. Taxonomy was assigned via SINTAX [22] using SILVA v138.2 as reference for the bacterial datasets. ii) The bioinformatics analysis began by demultiplexing and trimming raw ONT reads using Cutadapt 5.0. Forward barcodes were removed using a barcode FASTA file, allowing a maximum error rate of 10% and enabling reverse-complement matching. Primer sequences were then trimmed from both ends of the reads through sequential rounds of trimming to ensure complete removal. Following trimming, reads were filtered by length and quality: reads shorter than 1350 bp or longer than 1550 bp were discarded, and low-quality bases were removed from both ends using a Phred quality cutoff of Q20. The quality of the resulting reads was assessed with FastQC, and summary reports were generated for each sample. ONT reads were clustered with VSEARCH at 97% sequence identity. The centroid sequence of each cluster was retained together with its abundance information. Centroid sequences were then aligned to SILVA NR99 database (v138.2) using Minimap2 (v2.28) with the map-ont preset optimized for ONT data. The resulting SAM files were converted to BAM format, sorted, and indexed using Samtools (v1.21). Taxonomic classification was performed using a best-hit approach, in which each read was assigned to the taxon corresponding to the highest-scoring alignment against the reference database after applying specific quality filters: minimum mapping quality of 20, aligned length of at least 500 bp, and estimated sequence identity of at least 0.85. Taxonomic abundances were then reconstructed by assigning each retained centroid to its reference taxon, and weighing the assignment by the number of reads represented in that centroid. Relative abundances were finally calculated for each sample from the centroid-size-weighted taxonomic counts.

The code has been uploaded to GitHub and is available at the following address: https://github.com/edoardopasolli/16s_nanopore.

#### Bioinformatics for Illumina sequences

After shipment and sequencing, Novogene performed the bioinformatic analysis of the Illumina data. After sequencing, bioinformatic analysis of Illumina data was performed by Novogene using a QIIME1 (1.9.1) - based approach. Briefly, raw paired-end FASTQ files were merged using FLASH 1.2.11, followed by quality filtering (--min-overlap 10 --max-mismatch-densit 0.2) and chimera removal. Operational taxonomic units (OTUs) were clustered at 97% sequence similarity using UPARSE v7. Taxonomic annotation of representative OTU sequences was in-house performed by aligning them against the SILVA NR99 database (v138.2), using BLASTN. When multiple matches were obtained, alignments were ranked by lowest E-value, followed by highest bit score, percentage identity, and alignment length, and the top-ranked match was retained for each OTU. OTU tables were subsequently used for diversity analyses, including alpha and beta diversity metrics.

The raw sequencing data from both Illumina and ONT were uploaded to the Sequence Read Archive (SRA) from NCBI (https://www.ncbi.nlm.nih.gov/sra/docs) under the project number PRJNA1303859.

#### Cross-platform variant analysis

To complement the taxonomic comparisons, we performed isolate-level sequence analyses using the 11 previously characterized bacterial isolates for which Sanger 16 S rRNA sequences were available. For each isolate, the forward and reverse Sanger reads were processed with SeqKit, and the reverse read was reverse-complemented. A consensus sequence was then performed; when no consensus contig was obtained, the longest Sanger read was retained as the primary isolate-specific reference. ONT reads were recruited against the Sanger-derived reference using minimap2. Recruitment was first performed with stringent thresholds of ≥ 90% identity, an aligned reference length of at least 1000 bp, and mapping quality (MAPQ) ≥ 20. If no reads were recovered, a fallback criterion of ≥ 85% identity, aligned length ≥ 800 bp, and MAPQ ≥ 10 was applied. Coverage statistics were calculated with Samtools, and variants were called with BCFtools using mpileup thresholds of minimum mapping quality 20 and minimum base quality 10. The homologous Illumina locus was also recovered for each isolate. A reference-guided Illumina consensus was generated by mapping quality**-**filtered reads to the Sanger-derived reference with minimap2, followed by Samtools and BCFtools processing. The Illumina workflow shared the variant-calling thresholds used in the ONT analysis (minimum mapping quality = 20 and sequence identity ≥ 90%), whereas the ONT-specific read recruitment criteria based on minimum alignment length was not applied due to the shorter read length of Illumina sequencing data. Sequence-level comparisons were then performed for ONT versus Sanger, Illumina versus Sanger, and ONT versus Illumina. For each isolate, we computed the number of recruited reads, the number of filtered alignments, mean depth, breadth of coverage, and the number of detected sequence variants in each pairwise comparison. Sequence variants included consensus-level SNPs and indels relative to the reference sequence. Summary statistics were averaged across isolates and reported separately for each metagenome.

### Data analysis

The R statistical environment (R version 4.1.2) was used for data analysis using RStudio [23]. Microbial community data from both ONT and Illumina sequencing were organized and analysed with R package phyloseq [24] and vegan 2.5-6 [25]. Rarefaction curves were generated with the rarecurve() function from the vegan R package. Sequencing depth across samples was standardized by rarefaction to the minimum sample read count and expected species richness at this depth was estimated using rarefy(). Additionally, stochastic rarefaction was performed with rarefy() to evaluate variability in richness estimates. Alpha diversity was assessed with Shannon diversity. The Shannon-Weaver index (H) is calculated as follows: H = -sum pi * ln pi, where pi is the proportional abundance of species i which is the default index calculated with diversity() function of vegan package. Additionally, from the Shannon-Weaver index, the diversity was calculated as follows: D = exp(H) [26]. Beta diversity was visualized by principal coordinate analysis (PCoA) based on Bray-Curtis dissimilarities. Bar-plots were generated using R packages phyloseq 1.38.0 [24] and ggplot2 3.4.2 [27].

## Results

The ONT sequencing of the 16 S rRNA whole fragment generated up to 5.4 Gb of high‑quality data. The preliminary analysis and quality check of the sequences obtained with the MinION device resulted in 3,908,291 sequences of approximately 1500 bp as reported in the FastQC report (Supplementary Material 2). The report shows the “Per base sequence quality” of the 16 S long-read sequencing data, indicating a variable quality distribution along the read length, with the high-quality scores remaining from 50 up to 1650 bp and decreasing at both the start and end of the reads.

Workflow (i) produced limited taxonomic resolution, yielding only 42 taxa prior to clustering and 9 taxa after, whereas the tailored bioinformatic workflow (ii) resulted in 5,270 taxa prior to clustering and 1,940 after (Table 1). ONT data showed strong variability depending on the bioinformatic workflow. When processed through the Illumina-oriented workflow (i), ONT sequence counts ranged from 471 to 545 in soil-biomass samples and from 109,779 to 285,459 in bulk-plate samples prior to clustering. After clustering, these values were reduced, ranging from 464 to 540 OTUs in soil-biomass samples and from 104,301 to 269,237 OTUs in bulk-plate samples (Table 1). The tailored ONT workflow (ii) increased the number of recovered sequences for all samples, yielding between 414,695 and 493,126 reads in soil-biomass samples and between 536,602 and 819,914 reads in bulk-plate samples prior to clustering (Table 1). Following clustering, the number of retained OTUs ranged from 129,817 to 141,680 in soil-biomass samples and from 136,727 to 186,149 in bulk-plate samples (Table 1), remaining higher than those obtained with workflow (i). Although the number of sequences varied substantially across workflows and processing steps, the tailored ONT workflow recovered a considerably higher taxonomic diversity than workflow (i), with 1,940 versus 9 taxa detected after clustering step.

**Table 1:** Number of ONT sequences obtained for each sample before and after clustering using bioinformatic workflows (i) and (ii). The last row reports the total number of unique taxa detected across all samples for each workflow and processing step

	workflow (i)		workflow (ii)	
	Pre-clustering	Post-clustering	Pre-clustering	Post-clustering
Soil_biomass1	545	527	425,404	129,817
Soil_biomass2	471	464	414,695	139,146
Soil_biomass3	543	540	493,126	141,680
Bulk_plate1	162,286	155,167	607,012	186,149
Bulk_plate2	285,459	269,237	819,914	154,043
Bulk_plate3	109,779	104,301	536,602	136,727
Taxa	42	9	5270	1940

Moreover, the rarefaction curves analysis (Fig. 1) further demonstrated the standard approach inefficiency (workflow (i); Fig. 1B). The biomass samples, which in the Illumina sequencing separated from the bulk plate samples (Fig. 1A), failed to act in the same way with the ONT sequencing showing fewer OTUs as well as number sequences (Fig. 1B). However, the application of the alternative tailored for long-read bioinformatics workflow (ii) led to improved results. Indeed, rarefaction curve analyses showed a similar trend for both Illumina and MinION sequencing (Fig. 1A - C).

**Fig. 2:**
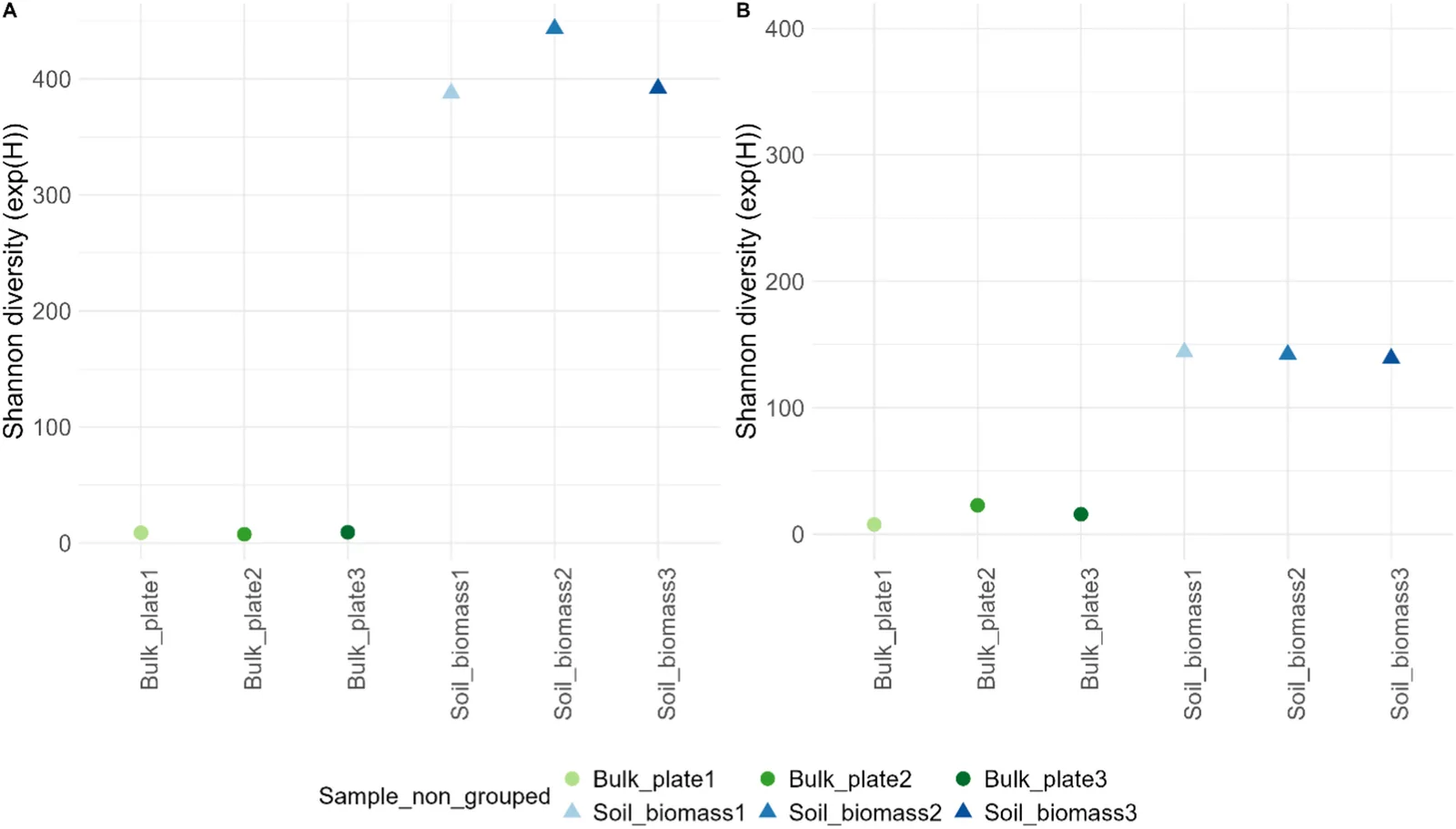
Alpha diversity of bulk plate (green) and mixed soil-lignocellulosic biomass (blue) samples of (**A**) data obtained with Illumina sequencing, (**B**) data obtained from MinION sequencing and analysed with tailored bioinformatic workflow (ii)

Shannon diversity was used to assess alpha diversity in bulk plate and mixed soil-lignocellulosic biomass, using data from Illumina sequencing and from ONT MinION analyzed with tailored workflow (ii). In the Illumina dataset (Fig. 2A), the bulk plate samples (green) displayed consistent lower alpha diversity values across the samples. Similarly, in the MinION dataset (Fig. 2B), the soil-biomass samples again demonstrated higher alpha diversity compared to the bulk plate samples, with a comparable trend observed across samples. While the absolute effective Shannon diversity values differed between the two sequencing platforms, the overall pattern of higher diversity in soil-biomass samples compared to bulk plate was consistently observed across both approaches.

**Fig. 3:**
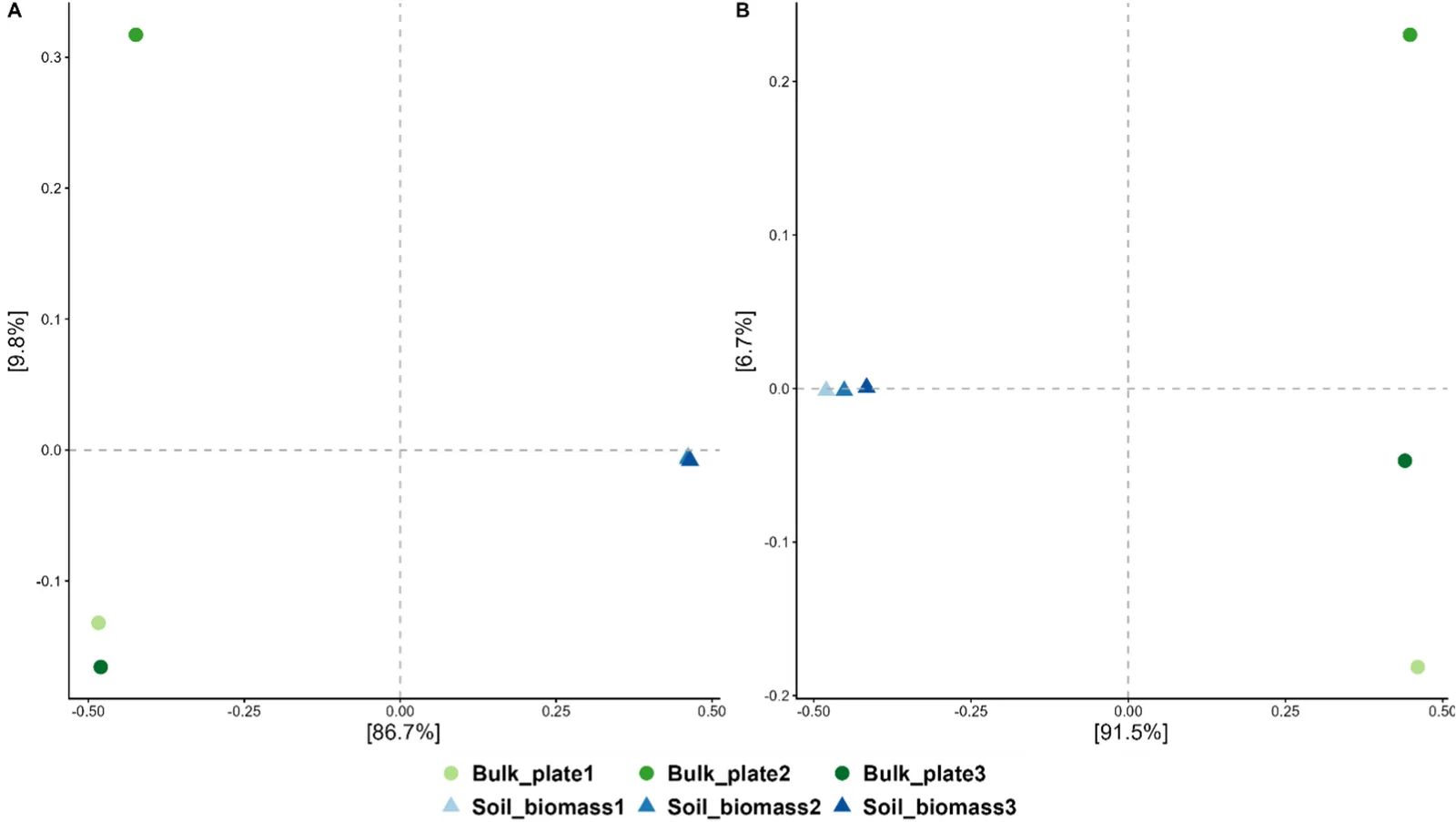
Beta diversity assessed with Bray-Curtis distance of bulk plate (green) and mixed soil-lignocellulosic biomass (blue) samples of (**A**) data obtained with Illumina sequencing, (**B**) data obtained from MinION sequencing and analysed with tailored bioinformatic workflow (ii)

We assessed beta diversity using Bray-Curtis distance metrics to examine how community composition differed between bulk plate (green) and mixed soil-lignocellulosic biomass (blue) samples. In the Illumina dataset (Fig. 3A), the samples clustered distinctly according to sample type. The bulk plate samples formed a tight group, clearly separated from the soil-biomass samples, which also clustered closely together. This indicates both a high degree of compositional dissimilarity between the two sample types and consistency within replicates. The MinION dataset (Fig. 3B) showed a similar clustering pattern. The bulk plate samples grouped tightly and separately from the soil-biomass samples, mirroring the segregation seen with the Illumina data. The high percentage of variation explained by the first two axes (Axis.1 = 91.5%, Axis.2 = 6.7%; Fig. 3B) further supports the robustness of this separation.

**Fig. 4:**
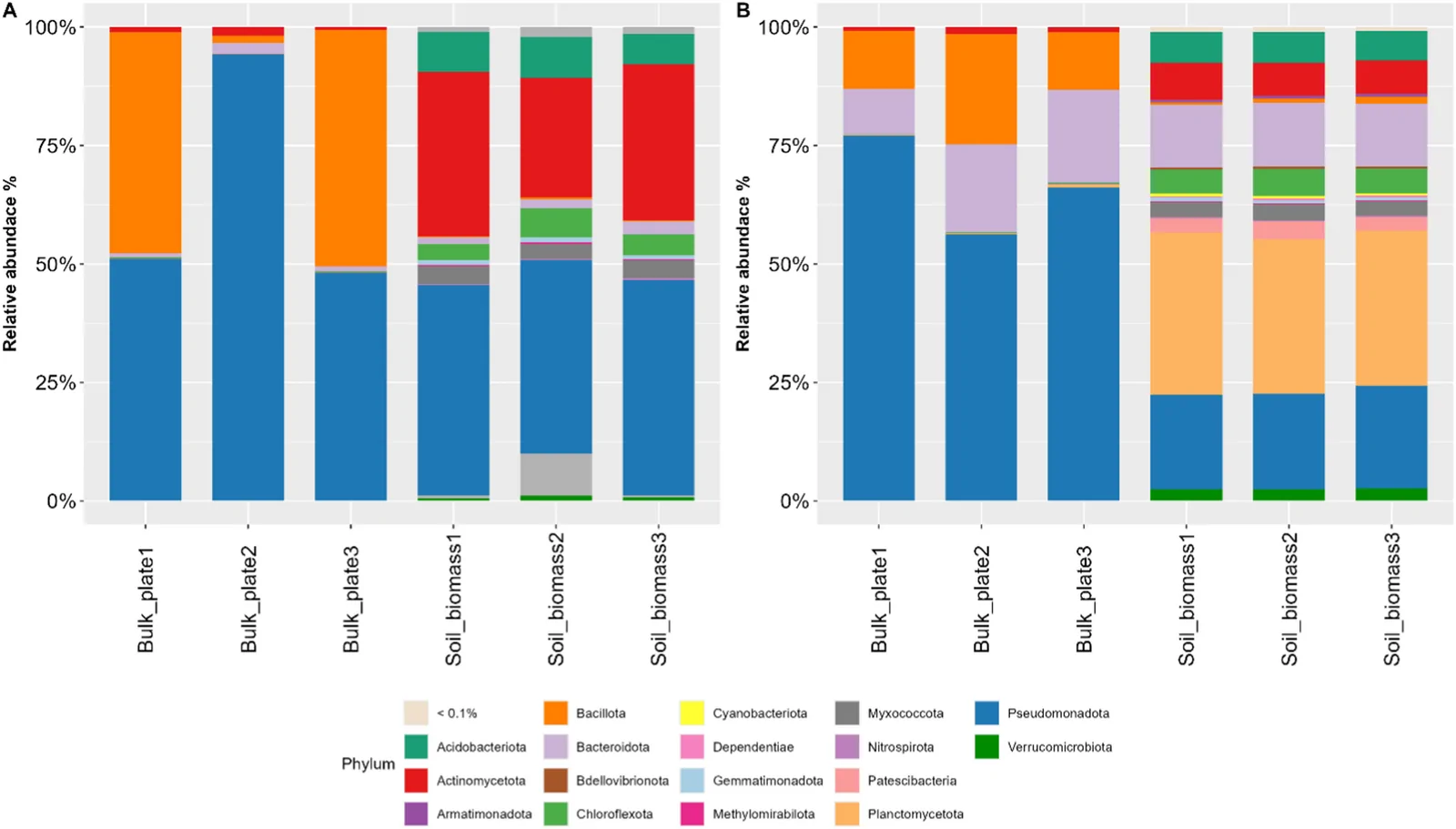
Bar plots showing the relative abundance of bacterial phyla of bulk plate and mixed soil-lignocellulosic biomass samples of (**A**) data obtained with Illumina sequencing, (**B**) data obtained from MinION sequencing analysed with tailored bioinformatic workflow (ii)

In the biomass samples, Illumina sequencing identified Pseudomonadota as the most abundant phylum, accounting for 43.51% of the total community, followed by Actinomycetota at 31.00%. Additional contributions were made by Acidobacteriota, Chloroflexota, Myxococcota, Crenarchaeota, and Bacteroidota; whereas other phyla such as Gemmatimonadota, Verrucomicrobiota, Nitrospirota, Methylomirabilota, Desulfobacterota, and Bacillota were detected at low relative abundances (< 1%, Fig. 4A). ONT sequencing of the same biomass samples also highlighted Pseudomonadota as the dominant phylum, although at a slightly lower relative abundance (29.28%, Fig. 4B). However, the ONT data revealed a more even distribution of taxa at the phylum level, with a broader representation of different phyla compared to Illumina data. Planctomycetota emerged as the second most abundant group in the ONT dataset (22.77%), while it was detected at low relative abundance in the Illumina data (~ 0.10% - Fig. 4B). Similarly, Bacteroidota (10.92%) and Actinomycetota (9.73%) were also well represented, along with Acidobacteriota, Chloroflexota, Bacillota, and Myxococcota. Several additional phyla, such as Patescibacteria and Verrucomicrobiota, were detected at intermediate levels, while others like Armatimonadota, Bdellovibrionota, and Dependentiae were present at low relative abundance (< 1%, Fig. 4B).

Bulk plate samples have a reduced microbial diversity, reflecting the selective nature of cultivation methods. Illumina data showed a strong dominance of Pseudomonadota at 64.35% and Bacillota at 32.65%, with only minor contributions from Bacteroidota, Actinomycetota, and Myxococcota (Fig. 4A). ONT sequencing of the same samples showed a comparable dominance by Pseudomonadota and Bacillota, at 53.07% and 37.19%, respectively. However, ONT revealed a slightly higher abundance of Bacteroidota and detected Actinomycetota at a similar level as Illumina. Although most remaining phyla occurred at very low levels, the presence of additional groups in trace amounts underscores the marginally higher sensitivity of ONT even in simplified microbial communities (Fig. 4B).

Taxonomic classification resolution improved notably when using ONT sequencing compared to Illumina (Table 2). At the phylum level, both platforms achieved high classification rates, with Illumina at 99.92% and ONT at 99.95% (Table 2). At the class level, ONT classified 99.13% of reads compared to 96.36% with Illumina; at the order level, 97.11% (ONT) vs. 88.21% (Illumina); and at the family level, 92.06%(ONT) vs. 71.53%(Illumina). The most substantial differences emerged at the genus and species levels, where ONT classified 82.47% and 49.38% of reads respectively, while Illumina classified only 35.32% and 4.59%. Illumina and ONT data produced distinct genus- and species-level assignments. Illumina reads, which were denoised and chimera-filtered, yielded more conservative taxonomic profiles compared to the ONT dataset. Conversely, ONT reads resulted in broader taxonomic representation, reflecting the distinct bioinformatic workflows applied to each sequencing platform.

**Table 2:** Percentage of annotated taxa at different taxonomic levels

	Illumina Sequencing (%)	ONT Sequencing (%)
Phylum	99.92	99.95
Class	96.36	99.13
Order	88.21	97.11
Family	71.53	92.06
Genus	35.32	82.47
Species	4.59	49.38

A total of 11 cellulolytic bacterial strains were previously isolated from CMC plates and identified at the species level by 16 S rRNA gene Sanger sequencing. These isolates were identified as follow *Bacillus* sp., FBB6AA; *Bacillus* sp., FBB6BA; *Microbacterium azadirachtae*, FBB6BB; *Bacillus* sp., FBB6BC; *Bacillus* sp., FBB7AA; *Bacillus pacificus*, FBB7AB; *Streptomyces* sp., FBB7BA; *Bacillus thuringiensis*, FBB7BB; *Dyella telluris*, FBB7BC; *Streptomyces chartreusis*, FBB8AA; *Paraburkholderia rhizosphaerae*, FBB8AB (Supplementary Material 1 Table 1). Isolation and bulk ONT sequencing revealed a strong correspondence at the genus level and, in some cases, at the species level. For *Bacillus*, the sequencing of bulk plates detected B. *thuringiensis* in all samples with 253–331–186 reads, while members of the *B. cereus* group (*B. cereus*,* B. mycoides*,* B. pseudomycoides*,* B. toyonensis*,* B. wiedmannii*) dominated with thousands of reads, reflecting the high 16S similarity within the clade. *Microbacterium azadirachtae* (FBB6BB) was detected in all bulk samples (29–20 − 17 reads), alongside minor contributions from other *Microbacterium* species. The genus *Dyella* (including *D. mobilis*,* D. terrae*,* D. marensis*) was consistently represented, though *D. telluris* (FBB7BC) was not assigned at the species level. *Streptomyces* isolates (FBB8AA and *S. chartreusis* FBB8AA) recovered at the genus level, but *S. chartreusis* itself was not detected. Finally, *Paraburkholderia rhizosphaerae* was not represented in the bulk sequencing data. Illumina amplicon data showed detection of the same taxa at the genus level (such as *Bacillus*, *Streptomyces* and *Dyella*).

**Fig. 5:**
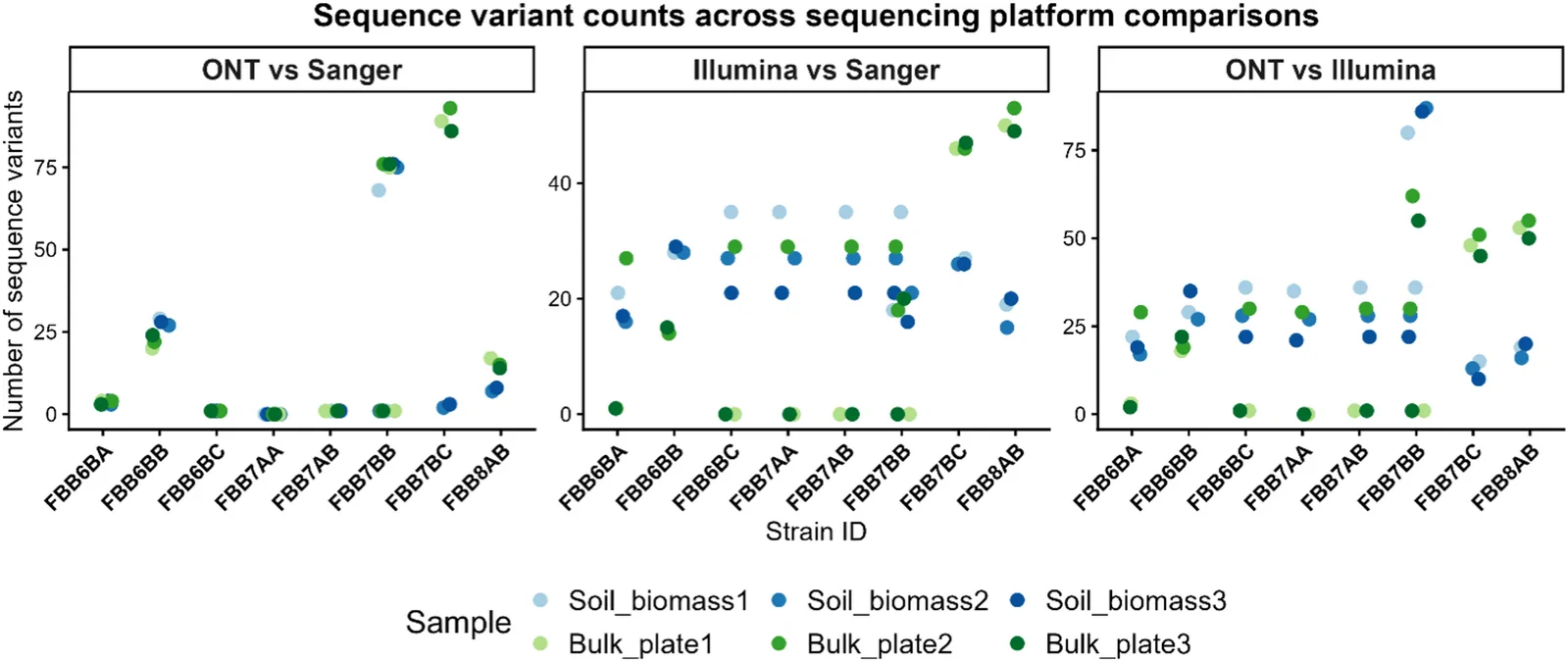
Sequence variant counts across sequencing platform comparisons (ONT vs. Sanger, Illumina vs. Sanger, and ONT vs. Illumina) for each bacterial strain. Points represent individual sample replicates from soil biomass (blue) and bulk plate (green)

Finally, to assess the consistency between sequencing platforms at the isolate level, sequence variants across ONT, Sanger, and Illumina data were compared (Fig. 5). Overall, ONT vs. Sanger comparisons showed low variant counts, indicating high concordance between the two methods. In several strains, including FBB6BA, FBB6BC, FBB7AA, and FBB7AB variant counts were consistently 0–1 across all replicates. In contrast, Illumina-based comparisons (Illumina vs. Sanger and ONT vs. Illumina) exhibited higher numbers of sequence variants, often across all replicates and sample types (Fig. 5).

## Discussion

Illumina NovaSeq and Oxford Nanopore Technologies (ONT) MinION are two sequencing platforms using distinct molecular techniques to determine DNA sequences in complex matrices. While ONT 16S workflows are less standardized than Illumina’s, recent bioinformatic tools are emerging. ONT remains more expensive per gigabase but offers competitive cost per sample, especially with multiplexing [3, 11].

In this study, we implemented a two-step PCR strategy combined with in-house barcoded primers (M13-tailed) to prepare 16S rRNA amplicons for long-read ONT sequencing using MinION.

This method allows multiplexing of samples without using the commercial ONT barcoding kit (SQK-PBK114). Based on reagent costs, the estimated per-sample cost for barcoding using custom M13 primers is approximately €3–5 ($3.3–5.5), compared to €12–15 ($13-16.5) per sample when using the ONT barcoding kit. Additional costs for PCR reagents (KAPA HiFi and SYBR FAST) and AMPure XP beads cleanup add roughly €1.70 ($1.9) per sample. The ligation and sequencing costs remain consistent (~€70–100 - $77–110) per sample, depending on the level of multiplexing. Overall, the total estimated cost per sample using this “homebrew” barcoding method is approximately €75 ($82.5), compared to €100–115 ($110–127) per sample using the standard ONT barcoding kit, yielding a potential savings of approximately 25–30% per sample [28, 29]. While these estimations demonstrate that the proposed workflow can reduce per-sample costs within the ONT framework, comparisons with alternative sequencing technologies require a broader perspective that considers throughput, infrastructure requirements, and sample processing capacity. Illumina’s NovaSeq 6000 remains one of the most widely used high-throughput platforms, because of its highly competitive costs, advanced infrastructure and large sample volumes. In contrast, Oxford Nanopore’s MinION, though more expensive per Gb offers portability and scalability ideal for smaller labs or field-based studies. Nevertheless, its higher per-sample cost could hinder its wider adoption, as the choice of sequencing platform is often influenced as much by cost as by performance.

Previous comparisons of microbial communities, such as those in dust [9] or agricultural soils [11], showed that the two platforms can produce different results, especially with notable differences at the genus and species levels. This study builds on prior work, comparing Illumina and ONT sequencing platforms by extending the analysis to analyse two very different samples, a simplified microbial community (bacterial bulk plates) and complex environmental samples (lignocellulosic biomass residues and soil). The focus was the application of ONT sequencing for species-level bacterial identification in complex systems with comparison with simpler samples (microbial cells collected from solid media: bulk plate) to evaluate its performances; our work also proposes a new bioinformatics workflow for long-read data, and introduces a cost-reduction strategy for ONT sequencing by developing a custom hybrid barcoding workflow (PacBio and ONT).

The preliminary results of the 16S rRNA long reads obtained with hybrid barcoding workflow and MinION sequencing yielded ~ 3.9 million long reads (~ 1500 bp) with a data output of ~ 5.4 Gb, confirming previous trends described for soil samples [30]. The FastQC report also showed a good per-sequence quality with a relatively high and stable quality between 50 and 1650 bp, suggesting that while the central portions of the reads are reliable, a quality trimming may be necessary to remove low-quality bases, to improve downstream analysis. Furthermore, the high read count and quality suggest a successful effective barcoding strategy, library preparation, a right amount of input DNA, along with a good flow cell quality. Karin et al. 2023 [31] demonstrated that PacBio’s Barcoded Universal Primers (BUPs) can be used to multiplex long amplicons across PacBio and ONT platforms but reported reduced ONT recovery (< 50% of input fragments), likely due to the primer design being optimized for PacBio sequencing chemistry. Importantly, the ONT data in their study were generated using earlier flow cell chemistry and basecalling models. Since then, recent improvements in ONT chemistry, pore design, and consensus calling have markedly enhanced amplicon sequencing performance, including homopolymer resolution and read accuracy.

Despite these encouraging results, analysing data with the standardized bioinformatics workflow used for Illumina application produced poor quality results, as previously observed [3, 11] underlying the need for bioinformatic protocol built ad hoc for long reads DNA sequences. However, the development and the application of a tailored bioinformatics workflow for long reads led to interesting results, comparable to Illumina data.

This study introduces a customized ONT sequencing workflow that offers multiple benefits over conventional workflows, especially for microbial profiling in complex environmental matrices. The approach enhances taxonomic resolution to the species level and substantially lowers library preparation costs by integrating in-house barcoding methods and full-length 16S rDNA sequencing, coupled with best-hit alignment via Minimap2 to the SILVA NR99 v138.2 database. Explicit primer removal and low-complexity filtering improve sequence quality and the accuracy of downstream taxonomic classification. The clustering-based analysis provided an internal validation of the results. Under a VSEARCH-based 97% clustering strategy followed by centroid classification and stringent alignment filtering. Although ASV-based denoising approaches are currently regarded as the standard strategy for Illumina metabarcoding analyses, OTU clustering was considered here for comparative purposes and to assess its applicability to ONT long-read datasets. Furthermore, the implementation of a new tailored workflow was supported by the need of a fully customizable approach. In particular, the proposed workflow allows the control of each analytical step, including read trimming, quality filtering, alignment stringency, and post-processing of mappings. This level of control is relevant for long-read ONT data, where parameter choices can substantially influence downstream taxonomic interpretation. By retaining full access to intermediate files and decisions, this approach facilitates reproducibility, method optimization, and critical evaluation of each processing stage. However, these benefits come with trade-offs. The workflow requires a higher level of bioinformatics expertise and manual customization, which may limit its accessibility for laboratories without specialized capabilities. As parameters need to be adjusted according to the user’s requirements, the current implementation should not be considered a solution for general use. Furthermore, the inherent error profiles of ONT long reads, if not properly addressed, can affect classification reliability. In addition, the computational requirements of the tailored workflow are depending on the dataset size. For the analysis of the soil samples in this study (*n* = 6), the workflow ran on a standard workstation using 8–16 GB of RAM and a multi-core CPU. Memory usage may increase with larger datasets or more complex reference databases. Despite these limitations, the method offers a flexible and cost-effective framework for high-resolution microbial community analysis and is appropriate for comparative studies alongside more traditional Illumina-based approaches.

Furthermore, the two sequencing platforms produce comparable trends when analysing rarefaction curves and alpha and beta diversity. The outputs in terms of number of sequences varied across platforms and bioinformatic analysis; even if, these values are strongly influenced by sequencing technology, throughput, and multiplexing strategies, and should not be interpreted as a direct comparison between technologies. These results highlight differences among processing workflows rather than to provide a direct quantitative comparison between the two sequencing platforms. Firstly, ONT produced sufficiently high sequencing depths to support rarefied alpha and beta diversity analyses comparable to Illumina. However, it is important to note that alpha diversity metric (e.g. Shannon Diversity), may underestimate true diversity if rarefaction or quality filtering results in the loss of reads - particularly from complex or uneven communities - potentially underestimating ecologically relevant patterns. Both platforms captured intra-group consistency and inter-group distinction, affirming ONT’s reliability. These results highlight the potential applications of the proposed method, in contrast to the findings of Stevens et al., 2023 [11], who observed different trends between the MiSeq and MinION performances especially considering alpha diversity.

The comparison of microbial community profiles obtained through Illumina and ONT sequencing reveals both overlapping patterns and key differences in the detection of dominant phyla across biomass and bulk plate samples. Illumina sequencing of biomass samples identified Pseudomonadota and Actinomycetota as the dominant phyla, with other groups present in lower abundances. In contrast, ONT sequencing also found Pseudomonadota to be dominant but revealed a more diverse and evenly distributed community. Planctomycetota and Bacteroidota were notably more abundant in ONT data, highlighting methodological differences in community profiling. Bulk plate samples exhibited reduced microbial diversity due to the selective nature of cultivation. Both Illumina and ONT sequencing revealed dominance by Pseudomonadota and Bacillota. ONT detected slightly higher levels of Bacteroidota and showed similar detection of Actinomycetota compared to Illumina. The simplified community structure facilitated comparison between platforms.

This trend across taxonomic ranks, resulting from the combination of ONT technology and long-read sequencing, where different primer sets, and a new bioinformatic approach resulted in the detection of a higher number of taxa compared to Illumina. The observed differences in the reported taxonomic profiles are not due to difference in DNA extraction methods -usually considered as the first main source of variability**-** as the same DNA template was used, but may be partially accounted for by the use of different primer sets which are optimized for each platform and introduces potential taxonomic biases [32, 33]. Otherwise PCR cycling conditions can be another source of variability as reported by Manter et al., 2024 [30]. Differences can be mainly attributable to variability in the PCR workflows rather than PCR contaminations, as negative controls assessed by agarose gel electrophoresis did not show visible bands. However, since these controls were not sequenced, the possibility of low-level contamination cannot be completely excluded. Furthermore, a point-source contaminant also appears unlikely given the consistency observed among replicates. Otherwise, relative abundance plots can be misleading when based on low-depth samples, as limited sequencing depth reduces the likelihood of detecting low-abundance taxa. Previous studies demonstrated that also bioinformatic processing, can influence taxonomic profiles, making challenging the comparisons across sequencing platforms [34]. Bioinformatic processing, along with the possibility of false positive identifications inherent to metabarcoding approaches, may contribute to the higher number of taxa detected [35]. Therefore, differences in PCR conditions, sequencing depth, bioinformatic processing, platform-related biases, and potential false-positive taxonomic assignments may contribute to the observed differences and cannot be fully solved within the present experimental design.

ONT sequencing showed improved taxonomic resolution at the genus and species levels, likely due to the full-length 16S reads, though results depend on base calling accuracy and classifier performance. While both technologies provide comparable results at the high taxonomic levels, ONT shows a marked improvement in annotating sequences at genus and species (Table 2) level, likely due to its ability to generate full-length 16 S sequences, which offer more comprehensive information for precise taxonomic classification. This enhanced capability allows for a more detailed understanding of microbial diversity and community structure within complex samples; even if estimated abundances derived from PCR-based amplicon sequencing are semi-quantitative, as they may be influenced by amplification biases and primer specificity. The higher annotation rates suggest that it is particularly valuable for studies requiring deeper taxonomic identification leading to more accurate interpretations of ecological and evolutionary relationships within bacterial communities. These findings are consistent with previous research supporting the adoption of long-read sequencing methodologies to study microbial ecology of complex samples such as mock microbial communities, dust, and soil [9, 36, 37].

In the present study, we did not explicitly benchmark barcode performance or include mock communities. Consequently, differences in taxonomic assignments between Illumina and ONT should be interpreted with caution. However, a comparison was performed between culture-dependent identification of cellulolytic bacteria and bulk sequencing approaches. A general agreement at the genus level was observed for both ONT and Illumina data, while ONT enabled species-level identification in some cases, which was not achieved with Illumina. This analysis revealed a concordance at the genus level. All genera represented by the isolated strains -including *Bacillus*, *Microbacterium*, *Dyella*, and *Streptomyces*-were detected in the sequenced bulk plate samples, demonstrating that MinION-based 16S rRNA gene sequencing can reliably capture the taxonomic composition of cellulolytic communities grown on CMC medium. Bulk ONT sequencing effectively recovered the major clades identified through isolation, particularly for *Bacillus* and *Microbacterium*, with quantitative reads supporting the presence and relative abundance of these taxa. Species-level assignment was successful for isolates with distinctive 16S sequences, such as *Microbacterium azadirachtae*, and *Bacillus thuringiensis*. Furthermore, the detection of *Dyella* at the genus level was consistent across bulk samples, underscoring ONT’s reliability for genus-level profiling even when species-level identification is challenging. In contrast, *Streptomyces* proved problematic, with some species, including *Streptomyces chartreusis*, undetected, consistent with known 16S ambiguities of this group. *Paraburkholderia* was not clearly represented, likely due to low abundance or competition effects in the bulk samples. Furthermore, Illumina amplicon data showed detection of the genera *Bacillus*, *Streptomyces* and *Dyella*, but species-level assignments were not achieved, potentially due to the short-read length and the limited discriminatory power of partial 16 S regions.

Furthermore, ONT and Illumina sequences were compared against isolate-specific Sanger sequences. Overall, ONT vs. Sanger comparisons showed low variant counts across most strains, indicating high sequence concordance. This pattern was consistent for several isolates (FBB6BA, FBB6BC, FBB7AA, and FBB7AB), supporting the reliability of ONT-based consensus reconstruction. However, notable strain-specific discrepancies were observed, particularly for *Dyella telluris* FBB7BC and *Bacillus thuringiensis* FBB7BB, which showed elevated variant counts. These differences likely reflect the presence of closely related taxa and the limited resolution of the 16 S rRNA gene. In contrast, Illumina-based comparisons yielded higher numbers of variants, even where ONT showed near-perfect agreement with Sanger. These observations strengthen the overall interpretation that the ONT full-length 16S workflow can provide reliable sequence information in simplified microbial communities when combined with an appropriate analysis approach. At the same time, it should be noted that these comparisons were limited to the isolates included in this study and, for Illumina, to cases where a homologous locus could be reliably recovered. Therefore, the sequence-level comparison should be interpreted as an internal validation of concordance within this experimental framework rather than as a universal benchmark of platform performance.

These findings highlight the complementary nature of ONT sequencing and culture-based approaches: bulk ONT provides a rapid and quantitative overview of community composition, while isolates remain critical for precise species-level characterization. The use of previously characterized isolates as a reference provides an internal validation of the ONT workflow, demonstrating its reliability for community-level analyses of bulk samples.

## Conclusion

This study supports the potential of ONT long-read sequencing for profiling complex microbial communities, especially when combined with a custom bioinformatic workflow. Compared to Illumina, ONT offers improved taxonomic resolution at the genus, and, in some cases, species level compared to short-read, depending on effective error correction and tailored analytical methods. Otherwise, another, primary advantage of this approach is the cost reduction and flexibility in primer design, while maintaining compatibility with ONT library preparation and high-accuracy sequencing. Such cost optimization is particularly relevant for studies requiring high sample throughput or pilot experiments where the use of commercial barcoding kits may represent a significant proportion of the overall sequencing budget. The integration of ONT sequencing with a customized bioinformatic workflow, developed specifically for this study, was essential to fully exploit the potential of full-length 16S rRNA reads. Unlike conventional Illumina workflows, which assume short, high-accuracy reads and are not directly compatible with ONT data, the tailored workflow allowed for improved species-level classification. It also enabled the detection of a broader range of taxa, though the identification of low-abundance phyla must be interpreted with caution due to ONT’s inherent error profile. These findings underscore the importance of using dedicated analytical tools specifically adapted to long-read sequencing data for accurate taxonomic assignments. Furthermore, future implementations of the tailored workflow could incorporate a Lowest Common Ancestor (LCA) approach to assign sequences with multiple near-equivalent matches to the deepest shared taxonomic rank. Additionally, a cost-effective hybrid barcoding strategy based on PacBio-compatible adapters significantly reduced library preparation costs without compromising sequencing performance. While this workflow requires greater technical and computational expertise, it offers a flexible and affordable option for high-resolution microbial community analysis in environmental and other research contexts such as clinical, and food microbiology.

## Supplementary Information

Supplementary Material 1.

Supplementary Material 2.

## Data Availability

The raw sequencing data were uploaded to the Sequence Read Archive (SRA) from NCBI (https://www.ncbi.nlm.nih.gov/sra/docs) under the project number PRJNA1303859.
