# Supplementary material for: A hybrid and cost-efficient barcoding strategy for full-length 16S rRNA gene nanopore sequencing of environmental samples
Source: BMC Genomics. 2026 Aug 10;27:741. doi: 10.1186/s12864-026-13239-z (PMC13551857; doi:10.1186/s12864-026-13239-z)
Supplement: Supplementary file 1 [file 12864_2026_13239_MOESM1_ESM.docx]

**A hybrid and cost-efficient barcoding strategy for full-length 16s rRNA nanopore sequencing of environmental samples**

**Ida Romano^1*^, Edoardo Pasolli^1,2^, Jean-Claude Walser^3^, Valeria Ventorino^1,2^, Sonja Reinhard^4^, Giuseppina Magaraci^1^, Olimpia Pepe^1,2^, Natacha Bodenhausen^4*^**

*^1^ Department of Agricultural Sciences, Division of Microbiology, University of Naples Federico II, Naples, Piazza Carlo di Borbone 1, Portici (NA), Italy*

*^2^ Task Force on Microbiome Studies, University of Naples Federico II, Naples, Italy*

*^3^ ETH Zurich Genetic Diversity Centre, Universitätstrasse 16, Zurich, Switzerland.*

*^4^ Department of Soil Sciences, Research Institute of Organic Agriculture (FiBL), Ackerstrasse 113, Frick, Switzerland*

** Corresponding authors:* Natacha Bodenhausen (e-mail: natacha.bodenhausen@fibl.org); Ida Romano (e-mail: ida.romano@unina.it)

**Supplementary material 1** Quality assessment of ONT sequencing data generated using FastQC, including per-base sequence quality, GC content, sequence duplication levels, and adapter content.

*See supplementary file : Supplementary Materials_1*

**Supplementary table 1** Molecular identification of bacterial isolates based on sequence similarity (% identity) to reference sequences, showing isolate IDs, closest matched taxa, and corresponding locus accession numbers.

| **Identification (% identity)** | **ID** | **LOCUS** |  |
| --- | --- | --- | --- |
|  |  |  |  |
| *Bacillus* sp. (96%) | **FBB6AA** | *PX777770* |  |
| *Bacillus* sp. (96%) | **FBB6BA** | *PX776785* |  |
| *Microbacterium* *azadirachtae* (99%) | **FBB6BB** | *PX777775* |  |
| *Bacillus* sp. (96%) | **FBB6BC** | *PX777776* |  |
| *Bacillus* sp. (96%) | **FBB7AA** | *FBB7AA* |  |
| *Bacillus* *pacificus* (100.00%) | **FBB7AB** | *PX777787* |  |
| *Streptomyces* sp. (96%) | **FBB7BA** | *N/A* |  |
| *Bacillus* *thuringiensis* (100%) | **FBB7BB** | *PX777875* |  |
| *Dyella* *telluris* (99%) | **FBB7BC** | *PX776699* |  |
| *Streptomyces* *chartesuis* (99%) | **FBB8AA** | *N/A* |  |
| *Paraburkholderia* *rhizosphaerae* (99%) | **FBB8AB** | *PX776818* |  |

*N/A: locus not yet assigned (submission in progress).*

**Supplementary Figure 1.** Representative agarose gel electrophoresis of 16S rRNA gene amplicons. Lane 1, 1 kb DNA ladder, with the smallest fragment corresponding to 500 bp; lanes 2 - 7, samples amplified using 1 µL of undiluted DNA: Soil_biomass1, Soil_biomass2, Soil_biomass3, Bulk_plate1, Bulk_plate2, and Bulk_plate3, respectively; lanes 9 - 14, the same samples amplified using 1 µL of DNA diluted 1:10, in the same order; lane 15, no-template control (NTC).
