# Supplementary material for: A hybrid and cost-efficient barcoding strategy for full-length 16S rRNA gene nanopore sequencing of environmental samples
Source: BMC Genomics. 2026 Aug 10;27:741. doi: 10.1186/s12864-026-13239-z (PMC13551857; doi:10.1186/s12864-026-13239-z)
Supplement: Supplementary file 2 [file 12864_2026_13239_MOESM2_ESM.html]

bamboo22.fastq.gz FastQC Report 

FastQC Report

Fri 2 Feb 2024  
bamboo22.fastq.gz

## Summary

- Basic Statistics
- Per base sequence quality
- Per sequence quality scores
- Per base sequence content
- Per sequence GC content
- Per base N content
- Sequence Length Distribution
- Sequence Duplication Levels
- Overrepresented sequences
- Adapter Content

## Basic Statistics

| Measure | Value |
| --- | --- |
| Filename | bamboo22.fastq.gz |
| File type | Conventional base calls |
| Encoding | Sanger / Illumina 1.9 |
| Total Sequences | 3908291 |
| Sequences flagged as poor quality | 0 |
| Sequence length | 500-2500 |
| %GC | 52 |

## Per base sequence quality

## Per sequence quality scores

## Per base sequence content

## Per sequence GC content

## Per base N content

## Sequence Length Distribution

## Sequence Duplication Levels

## Overrepresented sequences

| Sequence | Count | Percentage | Possible Source |
| --- | --- | --- | --- |
| ATGTCCTGTACTTCGTTCAGTTACGTATTGCTGGTAGTATATACAGAGTC | 12074 | 0.3089329837517217 | No Hit |
| TTGTCCTGTACTTCGTTCAGTTACGTATTGCTGGTAGTATATACAGAGTC | 7771 | 0.19883371018176488 | No Hit |
| ATGCCCTGTACTTCGTTCAGTTACGTATTGCTGGTAGTATATACAGAGTC | 7205 | 0.1843516769861814 | No Hit |
| ATGTACTTCGTTCAGTTACGTATTGCTGGTAGTATATACAGAGTCGAGCA | 6716 | 0.17183981438434345 | No Hit |
| TGTCCTGTACTTCGTTCAGTTACGTATTGCTGGTAGTATATACAGAGTCG | 4977 | 0.1273446629230014 | No Hit |
| ATGTACTGTACTTCGTTCAGTTACGTATTGCTGGTAGTATATACAGAGTC | 4765 | 0.12192029713242951 | No Hit |
| TGTACTTCGTTCAGTTACGTATTGCTGGTAGTATATACAGAGTCGAGCAG | 4648 | 0.11892666129517991 | No Hit |
| ATGTCCTGTACTTCGTTCAGTTACGTATTGCTGGTAGCTCACTACGCGCG | 4261 | 0.10902463506427745 | No Hit |
| ATGTGCCTGTACTTCGTTCAGTTACGTATTGCTGGTAGTATATACAGAGT | 4068 | 0.10408641526437003 | No Hit |

## Adapter Content

Produced by FastQC (version 0.11.9)
